# The role of endogenous interleukin-2 in proliferation of human carcinoma cell lines

**DOI:** 10.1038/sj.bjc.6690770

**Published:** 1999-11

**Authors:** T E Reichert, Y Kashii, J Stanson, A Zeevi, T L Whiteside

**Affiliations:** Departments of Pathology andOtolaryngology, University of Pittsburgh School of Medicine andUniversity of Pittsburgh Cancer Institute, Pittsburgh, PA, USA

**Keywords:** IL-2, human carcinomas, tumour cell growth, IL-2/IL-2R pathway, antisense IL-2 oligonucleotides, apoptosis

## Abstract

Interleukin (IL-2) and IL-2Rβ/γ have been shown to be expressed in human carcinomas in culture and in situ. Recently, expression of endogenous IL-2 and IL-2R in the cytoplasm was found to be up-regulated in tumour cells undergoing mitosis. This observation suggested that similar to its role in lymphocytes, the IL-2/IL-R pathway is involved in the regulation of carcinoma cell proliferation. Metabolic labelling followed by immunoprecipitation and Western blot results showed that IL-2 in carcinomas was identical to that in human lymphocytes. However, tumour cells did not secrete IL-2 detectable by immunoassays, although membrane-associated IL-2 was detectable on a proportion of these cells cultured in the absence of exogenous IL-2. Antibodies to IL-2 failed to inhibit proliferation of carcinoma cells, but antibodies specific for the ligand-binding site of the IL-2R were growth inhibitory. Growth of tumour cells was also inhibited by the immunosuppressive drugs, cyclosporin A (CsA), FK506 and rapamycin (RPA), known to interfere with the IL-2 pathway in lymphocytes. To further confirm the role of endogenous IL-2 in the growth of carcinomas, tumour cells were incubated with an IL-2-specific antisense oligonucleotide. The treatment was shown to transiently inhibit IL-2 mRNA and IL-2 protein expression as well as proliferation of tumour cells. Tumour cells treated with IL-2-specific antisense oligonucleotide demonstrated increased apoptosis in comparison to untreated or sense oligonucleotide-treated control cells. The data indicate that in human carcinomas, endogenous IL-2 promotes growth and protects tumour cells from apoptosis. © 1999 Cancer Research Campaign


*British Journal of Cancer* (1999) **81**(5), 822–831
© 1999 Cancer Research Campaign
Article no. bjoc.1999.0770

### The role of endogenous interleukin-2 in proliferation of human carcinoma cell lines

**TE Reichert[3]*, Y Kashii[3], J Stanson[3], A Zeevi[1] and TL Whiteside[1,2,3]**

Departments of [1]Pathology and [2]Otolaryngology, University of Pittsburgh School of Medicine and [3]University of Pittsburgh Cancer Institute, Pittsburgh, PA, USA

**Summary** Interleukin (IL-2) and IL-2Rβ/γ have been shown to be expressed in human carcinomas in culture and in situ. Recently, expression of endogenous IL-2 and IL-2R in the cytoplasm was found to be up-regulated in tumour cells undergoing mitosis. This observation suggested that similar to its role in lymphocytes, the IL-2/IL-R pathway is involved in the regulation of carcinoma cell proliferation. Metabolic labelling followed by immunoprecipitation and Western blot results showed that IL-2 in carcinomas was identical to that in human lymphocytes. However, tumour cells did not secrete IL-2 detectable by immunoassays, although membrane-associated IL-2 was detectable on a proportion of these cells cultured in the absence of exogenous IL-2. Antibodies to IL-2 failed to inhibit proliferation of carcinoma cells, but antibodies specific for the ligand-binding site of the IL-2R were growth inhibitory. Growth of tumour cells was also inhibited by the immunosuppressive drugs, cyclosporin A (CsA), FK506 and rapamycin (RPA), known to interfere with the IL-2 pathway in lymphocytes. To further confirm the role of endogenous IL-2 in the growth of carcinomas, tumour cells were incubated with an IL-2-specific antisense oligonucleotide. The treatment was shown to transiently inhibit IL-2 mRNA and IL-2 protein expression as well as proliferation of tumour cells. Tumour cells treated with IL-2-specific antisense oligonucleotide demonstrated increased apoptosis in comparison to untreated or sense oligonucleotide-treated control cells. The data indicate that in human carcinomas, endogenous IL-2 promotes growth and protects tumour cells from apoptosis. © 1999 Cancer Research Campaign

**Keywords:** IL-2; human carcinomas; tumour cell growth; IL-2/IL-2R pathway; antisense IL-2 oligonucleotides; apoptosis

The action of interleukin-2 (IL-2) on haematopoietic cells is mediated by its binding to the IL-2R complex, consisting of at least three distinct chains: α, β and γ (Waldmann, 1991; Nakanishi et al, 1992; Taniguchi and Minami, 1993). These three non-covalently associated chains form a high affinity IL-2R, with $K_d$ of $10^{-11}$ M. The IL-2R-α chain (p55) alone binds the ligand with a low affinity ($K_d = 10^{-8}$ M), and the IL-2Rβ chain (p70/75) in association with the γ chain (p64) is an intermediate affinity IL-2R with $K_d = 10^{-9}$ M (Arzt et al, 1992). IL-2-initiated signalling via IL-2R, necessary for lymphocyte activation and proliferation, is mediated primarily through the IL-2Rβ and γ chains, because the α chain lacks the cytoplasmic domain for signal transduction (Nakanishi et al, 1992).

Recent studies from our laboratory and others have demonstrated the expression of IL-2R, primarily IL-2Rβ and γ chains, as well as IL-2 in human tumour cells (Saneto et al, 1986; Arzt et al, 1992; Plaisance et al, 1992, 1993; Weidmann et al, 1992; Alileche et al, 1993; Ciacci et al, 1993; Rimoldi et al, 1993; Yasamura et al, 1994; Lin et al, 1995; McMillan et al, 1995). These components of the IL-2 pathway were expressed in tumour cells grown in the absence of exogenous IL-2 as well as in tumour biopsy specimens (Weidmann et al, 1992; Lin et al, 1995). Our findings, which suggest that endogenous IL-2 might be involved in the regulation of tumour cell growth, include: (1) the localization of intracytoplasmic IL-2 to the Golgi complex; (2) increased expression of IL-2 mRNA and protein in mitotic tumour cells; and (3) the association between increased IL-2 expression and a decreased level of $p27^{Kip1}$ cyclin-dependent kinase (CDK) inhibitor in mitotic cells (Reichert et al, 1998*a*). Previous results from other laboratories have also suggested that, similar to its role in proliferation of haematopoietic cells, IL-2 may be a growth hormone for tumour cells (Alileche et al, 1993).

The detection of IL-2R and of intracytoplasmic IL-2 in human tumour cells and normal epithelial cells raises the possibility that a functional IL-2/IL-2R pathway may exist in tumour or tissue cells of non-haematopoietic origin. Previous observations from our laboratory indicated that the addition of exogenous IL-2 at high concentrations (in nM to μM quantities) or of antibodies (Abs) recognizing the IL-2 binding site on the IL-2Rβ chain expressed in carcinoma cell lines inhibited their growth (Rabinowich et al, 1992; Yasamura et al, 1994; Lin et al, 1995). In contrast, recent results showed that endogenous IL-2, produced by tumour cells in the absence of exogenous IL-2, behaved like a cell cycle-associated protein and was involved in promoting cell cycle progression in carcinomas (Reichert et al, 1998*a*). These data prompted us to investigate the role of endogenous IL-2 in tumour cell growth.

In this study, we have used immunological as well as molecular strategies to demonstrate that IL-2 produced by human carcinomas is not only involved in tumour cell growth but may also protect tumour cells from apoptosis.

*Received 8 January 1999*
*Revised 17 May 1999*
*Accepted 17 May 1999*

*Correspondence to:* TL Whiteside, University of Pittsburgh Cancer Institute, 211 Lothrop Street, W1041 BST, Pittsburgh, PA 15213, USA

*Present address: Johannes Gutenberg-Universitat, Augustusplatz 2, 55191 Mainz, Germany

### MATERIALS AND METHODS

### Cell lines

Human squamous cell carcinoma of the head and neck (SCCHN) cell lines (PCI-1 and PCI-13) and the HR gastric carcinoma cell line have been established in our laboratory, as previously described (Heo et al, 1989; Shimizu et al, 1991). These lines were maintained in Dulbecco's modified-minimal essential medium (DMEM) containing 10% (v/v) heat-inactivated (56°C, 30 min) fetal calf serum (FCS), 2 mM glutamine, 100 U $ml^{-1}$ penicillin and 100 μg $ml^{-1}$ streptomycin (all from Life Technologies, Inc., Grand Island, NY, USA). The cells were passaged by trypsinization (0.25% trypsin solution, Life Technologies, Grand Island, NY, USA) and were routinely tested for mycoplasma (Gen-Probe, San Diego, CA, USA). For experiments described here, the cell lines were cultured in the same DMEM but supplemented with 1% (v/v) of FCS.

### Antibodies to human IL-2 and IL-2R chains

Unlabelled polyclonal and monoclonal antibodies (mAbs) to IL-2 were purchased from Genzyme, Cambridge, MA, USA, Becton Dickinson, Mountain View, CA, USA or R&D Systems, Minneapolis, MN, USA. Labelled mAbs to IL-2 were obtained from PharMingen, San Diego, CA, USA or Biosource International, Camarillo, CA, USA. Unlabelled mAbs to the α chain (CD25) of IL-2R were purchased from Dako Corporation, Carpinteria, CA, USA; and fluourescein isothiocyanate (FITC)-conjugated mAbs to CD25 from Becton Dickinson. The mAbs TU27 to the β chain of IL-2R was kindly provided by Dr Junji Hamuro (Ajinomoto Co., Inc., Kawasaki, Japan). The FITC-conjugated mAb to the β chain (anti-CD122) of IL-2R was obtained from Endogen (Boston, MA, USA) and unlabelled mAbs Mik-β2 and Mik-β3 to the β chain of IL-2R were purchased from PharMingen. Unlabelled antibodies to IL-2Rβ chain, which do not interfere with the ligand binding: 341 and 561, were generously provided by Dr Paul Sondel (University of Wisconsin, Madison, WI, USA). The polyclonal chicken Ab to the IL-2R-γ chain was purchased from Promega (Madison, WI, USA).

Labelled or unlabelled isotype control antibodies (IgG, IgM, $IgG_{2a}$) were purchased from Becton Dickinson, Dako Corporation, PharMingen or Biosource International. Isotype control for the IL2R-γ chain was chicken IgG purchased from Pierce, Rockford, IL, USA.

### Immunostaining for IL-2 and IL-2R

Tumour cell monolayers were grown to confluence (70–80%) on Labtek chamber slides (CMA, Houston, TX, USA). The slides were rinsed in cold phosphate-buffered saline (PBS) (2 × 3 min). The cells were pre-fixed for 5 min in 2% (w/v) paraformaldehyde (PFA) and then permeabilized with 0.1% Triton X-100 in PBS for 10 min before staining with mAbs. Indirect immunoperoxidase (IP) method (Chen et al, 1987) was used for staining with anti-IL-2 Abs (30 μg $ml^{-1}$). Prior to staining, polyclonal anti-IL-2 Ab was preincubated with excess rIL-2 (Chiron Corp., Emeryville, CA, USA) to verify its specificity. Aliquots (300 μl) of the Ab were mixed with equal volumes of rhIL-2 ($1.5 \times 10^6$ Cetus units $ml^{-1}$) and incubated at room temperature for 1 h. This preincubation completely eliminated cytokine-specific staining. Isotype control Abs (IgG mouse, IgG chicken and IgG rabbit) were used as appropriate. Slides were examined in an Olympus BH-2 light microscope to determine expression of IL-2 and IL-2R proteins in tumour cells.

### Flow cytometry

Expression of IL-2 and the different IL-2R chains on various tumour cell lines was determined by flow cytometry, using PE- or FITC-conjugated anti-IL-2, anti-CD25, anti-CD122 or unlabelled TU27 or anti-common γ chain Abs. For surface staining, tumour cell suspensions were adjusted to the concentration of $1 \times 10^6$ cells $ml^{-1}$ in 0.1% (v/v) sodium azide in PBS and incubated with pre-determined dilutions of appropriate Abs for 30 min at 4°C, washed and fixed with 1% (v/v) PFA-PBS. All Abs were pre-titred on normal non-activated or IL-2 activated PBMC to select optimal dilutions. To detect intracellular protein expression, tumour cells were first pre-fixed with 2% (w/v) PFA in PBS for 20 min at 4°C, washed twice, permeabilized with 0.1% saponin (Sander et al, 1991) and stained with appropriate Abs. For indirect staining, cells were incubated first with the primary Ab, washed three times and then incubated with the FITC-conjugated second Ab (goat anti-mouse or goat anti-chicken). The appropriate isotype IgG, chicken IgG or PBS alone were used as controls in all experiments.

### Transduction of tumour cells with the IL-2 gene

PCI-1 or HR tumour cell lines were stably transduced with the retroviral vector DFG-hIL-2-Neo, using supernatants of the packaging cell line, CRIP, selected in G418 medium and examined for the ability to secrete IL-2 using an enzyme-linked immunosorbent assay (ELISA), as previously described (Nagashima et al, 1997*a*, 1997*b*). As control, carcinoma cell lines were also transduced with the LacZ gene.

### ELISA for IL-2

Tumour cells were cultured, as described above, harvested, washed in PBS and subjected to a series of three freeze and thaw cycles to disrupt the tumour cells. Cytosol was diluted 1/4 with PBS and tested for the presence of IL-2 by ELISA (Endogen, Cambridge, MA, USA). The kit was calibrated against the WHO International Cytokine Standard. Interassay variability was monitored by the use of IL-2-rich supernatants which were prepared in bulk by activation of normal human peripheral blood mononuclear cells (PBMCs) with PHA-P and stored in small aliquots at –80°C. These supernatants were used as a positive control in every assay. The CV for IL-2 ELISA, established on the basis of 50 determinations, was 15 ± 3%.

### MTT proliferation assay

Cell lines were cultured in medium containing 1% (v/v) heat-inactivated (56°C, 30 min) FCS or in the presence of various concentrations of Abs, including TU27, Mikβ2, mAb 341, polyclonal rabbit anti-human IL-2 or mouse anti-human IL-2 mAb. Growth of these cells was measured using a colourimetric MTT [3-(4,5-dimethylthiazal-2-yl)-2,5-diphenyltetrazolium bromide] assay, as described by us previously (Lin et al, 1995). The OD readings at the wavelength of 540 nm were obtained from at least four wells per each concentration of every Ab used to modulate

© 1999 Cancer Research Campaign 

growth and the mean $OD_{540}$ ± s.d. calculated. Inhibition of proliferation in the presence of the Ab was calculated as the percentage of control cell growth, using the formula:

$$1 - \frac{\text{experimental OD}}{\text{control OD}} \times 100,$$

where control = OD of tumour cells cultured in medium alone.

### [$^3$H]-thymidine incorporation assay

To determine effects of cyclosporin A (CsA), FK 506 or rapamycin (RPA) on tumour cell growth, incorporation of [$^3$H]-thymidine into cellular DNA of HR, PCI-1 or PCI-13 cells in the presence of different concentrations of these drugs was measured. CsA was obtained from Sandoz (Basel, Switzerland), FK506 from Fujisawa Pharmaceuticals (Osaka, Japan) and RPA from Ayerst (Wyath Research, Princeton, NJ, USA). Tumour cell monolayers in 96-well, flat-bottomed plates ($5 \times 10^3$ well$^{-1}$) were incubated in tissue culture medium (control) or different dilutions of CsA, FK 506 or RPA in the absence if IL-2 for 24, 48 or 72 h at 37°C and 5% carbon dioxide in air. The cultures were pulsed with 1 μCi well$^{-1}$ of [$^3$H]-thymidine (specific activity: 1 Ci mM$^{-1}$; NEN, Boston, MA, USA) 6 h prior to harvest. Cells were washed three times, trypsinized, subjected to two freeze and thaw cycles, and harvested with a Skatron harvester (Tranby, Norway). Radioactivity was determined in a β counter. Results were calculated as the mean cpm of four replicate determinations and were expressed as per cent of control counts.

### Metabolic labelling and immunoprecipitation studies

Jurkat cells used as a positive control for these studies were activated by incubation in the presence of phytohaemagglutinin (PHA) at 1 μg ml$^{-1}$ and phorbol myristic acetate (PMA) at 50 ng ml$^{-1}$ for 24 h. For metabolic labelling, Jurkat cells or tumour cells were harvested, washed and placed in the methionine-free medium containing [$^{35}$S]-methionine (Amersham; sp. act > 1000 Ci mmol$^{-1}$) for 8 h. The cells were then lysed in the buffer containing 1% Triton X-100, 1 mM iodoacetamide, 0.2 U ml$^{-1}$ aprotinin and 1 mM phenylmethylsulphonyl fluoride (PMSF) prepared in TSA solution (0.01 M Tris–HCl, 0.14 M sodium chloride, 0.025% sodium azide). The lysates were precleared with Protein A-Sepharose (Sigma) and reacted overnight with polyclonal rabbit anti-IL-2 Ab (Genzyme) at the concentration of 1 μg Ab per 200 μl lysate. Samples were incubated for 2 h at 4°C with Protein-A Sepharose prior to centrifugation at 200 ***g*** for 2 min. Pellets were washed twice with 0.1% Triton X-100 prepared in TSA solution, then with TSA and, finally, with 0.05 M Tris–HCl pH 6.8. After washing, pellets were boiled for 5 min in sodium dodecyl sulphate (SDS)-sample buffer with 5% 2-mercaptoethanol and subjected to 15% SDS-polyacrylamide gel electrophoresis (SDS-PAGE). Gels were dried and exposed to film at –70°C for 24–96 h. To confirm specificity, 1 μg of anti-IL-2 Ab was absorbed with 20 μg of IL-2 (ratio of 20:1; Chiron) for 4 h at 4°C prior to its use for immunoprecipitation.

### Western blots

Cell lysates prepared from $1 \times 10^6$ carcinoma cells, Jurkat cells or YT, an NK-cell line, were electrophoresed on a gradient SDS polyacrylamide gel (4–20%) and then electrophoretically transferred to a nitrocellulose membrane. The nitrocellulose blot was blocked, washed and incubated with goat anti-human IL-2 Ab purchased from R&D Systems at a final concentration of 0.1 μg ml$^{-1}$. This process was repeated twice. The second antibody was labelled with horseradish peroxidase, and an enhanced chemiluminescence (ECL) system was used for detection.

### mRNA analysis by RT-PCR

Total RNA was extracted from tumour cells transduced with the IL-2 gene or with the lacZ gene as control, using a single step method with the RNAzol solution (Chomczynski and Sacci, 1987). RNA (100 ng) was recovered from each sample, transcribed and amplified by PCR as previously described (Nagashima et al, 1997*a*, 1997*b*).

The primers for IL-2 were as follows: sense: 5′-GTCA-CAAACAGTGCACCTAC-3′; antisense: 5′-CCCTGGGTCT-TAAGTGAAAG-3′; and the primers for β actin were: sense: 5′-GGGTCAGAAGGATTCCTATG-3′, antisense: 5′-GGTCT-CAAACATGATCTGGG-3′. For PCR, 35 cycles of amplifications were used with denaturing at 94°C for 1 min, annealing at 62°C for 1 min, and extension at 72°C for 1 min. The PCR was performed under conditions described previously (Nagashima et al, 1997*a*).

### Antisense oligonucleotide treatment

Based on the data indicating that IL-2 expressed in tumour cells was identical to that in lymphocytes, nucleotide sequences were selected in the 5′ coding region of hIL-2. The antisense and sense sequences were: 5′-CAGGAGTTGCATCCTGTACAT-3′ (ASO); 5′-ATGTACAGGATGCAACTCCTG-3′ (SO).

These nucleotides were phosphorothioated and purified by column chromatography in the DNA synthesis facility at the University of Pittsburgh. To study their uptake by tumour cells, the oligonucleotides were fluorescein-labelled, using the fluoro-Amp™ T4 kinase Green Oligonucleotide Labeling System (Promega), purified, adjusted to an appropriate DNA concentration and mixed with a cationic lipid transfection reagent N-[1-(2,3-dioleoyloxy)-propyl]-N,N,N-trimethyl ammonium methylsulphate (DOTAP, Boehringer Mannheim, Indianapolis, IN, USA). This reagent has been shown to enhance DNA uptake by cells and also to protect oligonucleotides from nucleolytic degradation within the cell (Degolos et al, 1991). Tumour cells were maintained in reduced serum (0.5–1%) medium to limit the degradation of oligonucleotides by serum-derived nucleases (Cappacioli et al, 1993). In preliminary experiments, cells were treated with DOTAP and labelled oligonucleotides at final concentrations of 5 μl DOTAP per 1 μg oligonucleotide for 24 h, washed free of the oligonucleotide in DOTAP-containing medium and examined in a fluorescent microscope for intracytoplasmic staining. In growth inhibition experiments, cells were plated at the concentration of $5 \times 10^3$ cells well$^{-1}$ in wells of a 96-well plate. On the following day, the cells were treated with various concentrations of oligonucleotides, ranging from 0.3 to 10 μM, complexed with DOTAP. Following, 24, 48 or 72 h incubation, growth of tumour cells was measured in an MTT assay, as described above.

### Apoptosis assay

Tumour cells, either PCI-1 or PCI-13, were cultured in the presence of medium containing IL-2 sense oligonucleotide (SO) or IL-2 antisense oligonucleotide (ASO) at a final concentration of

© 1999 Cancer Research Campaign

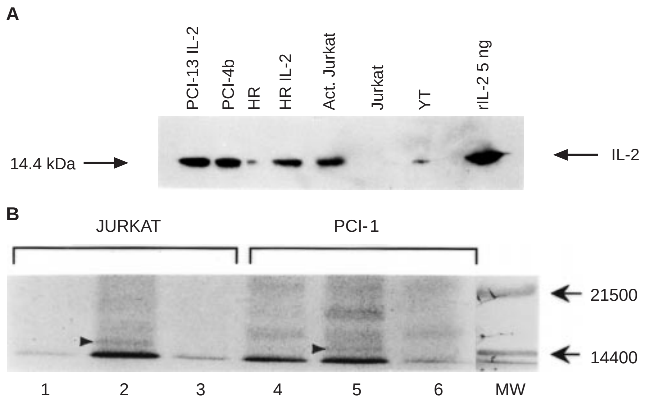

**Figure 1** Expression of IL-2 in human tumour cells. (**A**) Western blots of tumour cell lysates obtained from various tumour cells and of human rIL-2 (5 ng) used as control. Anti-human IL-2 Ab was used to visualize the 14.4 kDa band. Activated Jurkat and YT, an NK cell line, are used as positive controls and non-activated Jurkat as a negative control. PCI-13 IL-2 and HR-IL2 are tumour cells stably transduced with the hIL-2 gene. HR and PCI-4b are non-transduced carcinoma cell lines. (**B**) Metabolic labelling and immunoprecipitation of IL-2 in Jurkat cells (control) and PCI-1 tumour cells. Autoradiography following separation of cell lysates on PAGE was performed using samples precipitated in the presence of normal rabbit serum as control (lanes 1 and 4); of rabbit Ab to hIL-2 (lanes 2 and 5); or of rabbit Ab to hIL-2 absorbed with an excess of rIL-2 (lanes 3 and 6). The arrow heads show the position of the IL-2 band (14 400 kDA) in lanes 2 and 5. The difference in intensity of non-specific bands in lanes 5 and 6 is likely due to the use of unabsorbed versus absorbed Abs to hIL-2. Results of one experiment of three performed are shown

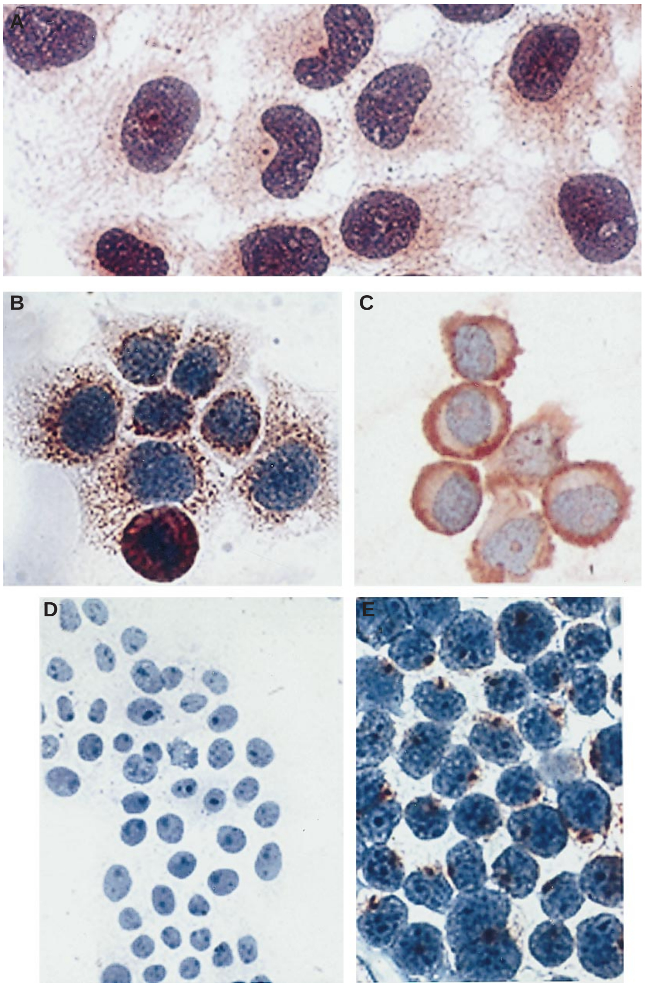

**Figure 2** Immunostaining of tumour cells for expression of IL-2 or IL-2R. (**A**) PCI-1 cells in a monolayer are immunostained for endogenous IL-2; Mag × 1000. (**B and C**) PCI-1 stained with anti-IL-2Rγ chain Ab or with TU27 Ab for IL-2Rβ Ab respectively; Mag × 1000. (**D**) PCI-1 cells are stained with isotype control Abs (negative control); Mag × 250. (**E**) Activated Jurkat T-cells are stained with anti-IL-2 Ab (positive control); Mag × 4000

1 μM, as described above, for 8, 24 or 48 h. Cells were harvested by trypsinization, washed and tested for: (a) the presence of endogenous IL-2 by flow cytometery; and (b) evidence of apoptosis, as assessed in TUNEL assays. The TUNEL assay was performed using reagents purchased from Boehringer Mannheim, as previously described by us (Whiteside et al, 1998).

### Statistical analysis

The results were analysed using the Mann–Whitney *U*-test. The differences were considered significant when $P$ values were $< 0.05$.

### RESULTS

### IL-2 expression in human carcinomas

The presence of IL-2 mRNA and protein in tumour cells in vitro and in situ has been previously demonstrated in our laboratory (Lin et al, 1995; Reichert et al, 1998*a*, 1998*b*). Also, endogenous IL-2 was detectable in the tumour cell cytosol by immunoassays at relatively low levels of 40–60 pg $ml^{-1}$ per $5 \times 10^6$ cells disrupted by rapid freezing and thawing (Lin et al, 1995). To confirm the presence of IL-2 in carcinoma cells, tumour lysates were prepared and Western blots performed, using anti-human IL-2 Ab and human recombinant IL-2 (Chiron) as a positive control (Figure 1A). This Western blot analysis showed that the 14.4-kDa band detected in tumour lysates by anti-IL-2 Ab corresponded to that obtained with rIL-2. Next, metabolic labelling with [$^{35}$S]-methionine followed by immunoprecipitation was performed, using PCI-1 cells and activated Jurkat cells as a positive control. As indicated in Figure 1B, the bands corresponding to IL-2 on the gel were identical for tumour-derived and lymphoid cell-derived IL-2. Furthermore, absorption with anti-IL-2 Abs eliminated the IL-2 band (Figure 1B), confirming that it corresponded to immunoprecipitated IL-2. In addition, immunoprecipitation experiments utilizing goat anti-human IL-2 Abs or isotype IgG control and performed with lymphoid or tumour cell lysates confirmed expression of the 14.4-kDa IL-2 band (data not shown). These experiments demonstrated that endogenous IL-2 in tumour cells was identical to that expressed in and produced by normal lymphoid cells.

Metabolic labelling of tumour cells allowed for the detection of IL-2 in concentrated (20×) supernatants of these cells (data not shown). The finding indicated that very small quantities of IL-2 were released by tumour cells. However, in numerous experiments, we have failed to detect IL-2 in unconcentrated supernatants of the same tumour cells by ELISA, in spite of abundant expression of IL-2 in the Golgi zone of these cells, as illustrated in Figure 2A. IL-2 expression in tumour cells was similar to that usually seen for IL-2 in activated Jurkat cells (Figure 2E). In biological assays, using an IL-2-dependent CTLL cell line, minimal levels of IL-2 were detectable in tumour cell supernatants

*© 1999 Cancer Research Campaign*

**Table 1** Effects of CsA, FK506 and RPA on growth of tumour cells[a]

| | HR | PCI-1 | PCI-13 |
|---|---|---|---|
| **Treatment** | **% Growth control** | | |
| CsA-24 h (1 μg ml$^{-1}$) | 58 ± 5* | 60 ± 7* | 63 ± 4* |
| CsA – 72 h (1 μg ml$^{-1}$) | 75 ± 3* | 40 ± 8* | 80 ± 5* |
| FK – 24 h (0.5 μg ml$^{-1}$) | 65 ± 5* | 63 ± 4* | 65 ± 4* |
| FK – 72 h (0.5 μg ml$^{-1}$) | 70 ± 4* | 80 ± 3* | 75 ± 4* |
| RPA – 24 h (10 μg ml$^{-1}$) | 83 ± 4* | 86 ± 6 | 85 ± 4 |
| RPA – 72 h (10 ng ml$^{-1}$) | 40 ± 5* | 60 ± 2* | 51 ± 6* |

[a]Proliferation of HR, PCI-1 and PCI-13 cells incubated in the presence or absence of the immunosuppressive drugs was measured following 24 h (short-term) or 72 h (long-term) incubation. Growth of tumour cells was measured in [$^3$H]-thymidine incorporation assays. The doses of the drugs were selected to give about 40–50% growth inhibition relative to medium controls. The data are mean values ± s.d. of four replicates. A representative experiment of 3 performed is shown. The asterisks indicate significant inhibition of proliferation ($P < 0.05$) compared to control cells incubated with medium alone.

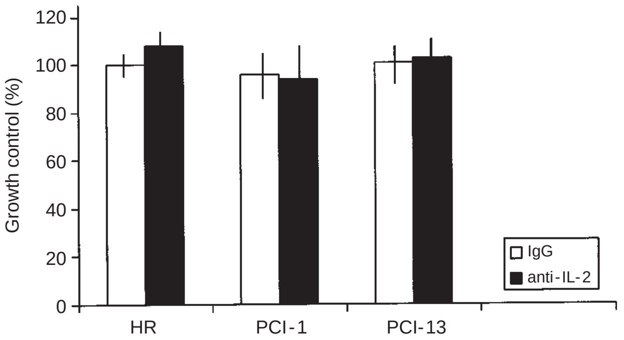

**Figure 3** Proliferation of HR, PCI-1 and PCI-13 cells incubated for 3 days in the presence of Abs to IL-2 at the concentration of 1 mg ml$^{-1}$. Proliferation was not inhibited at this or other Ab concentrations tested (0.01–10 mg ml$^{-1}$). Proliferation was measured in 3-day MTT assays. The data are presented as the % of cell growth in medium and are means ± s.d. of three replicate wells in a representative experiment of four performed. Isotype control IgG was used as control

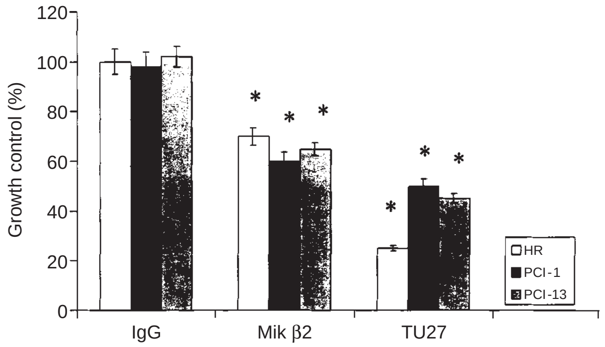

**Figure 4** Inhibition of growth of tumour cells in the presence of anti-IL-2Rβ mAbs Mikβ2, TU27 or control IgG. On day 3, growth of tumour cells was measured in MTT assays as described in Materials and Methods. The data are mean values ± s.d. of four replicates in a representative experiment of at least 3 performed. The asterisks indicate significant inhibition in proliferation ($P < 0.05$) compared to control cells incubated with isotype control IgG

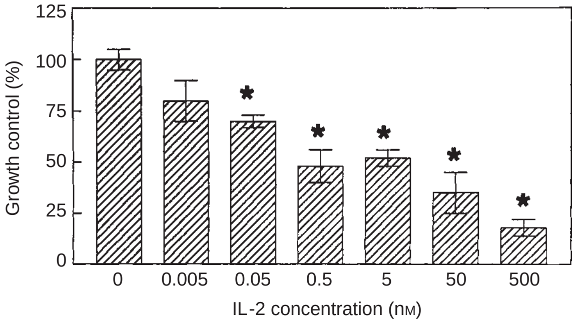

**Figure 5** Inhibition of tumour cell (PCI-13) proliferation by exogenous recombinant (r)IL-2. Tumour cells were incubated with various concentrations of human rIL-2, and their growth was measured in [$^3$H]-TdR incorporation assays. The data are presented as mean ± s.d. percentages of their growth relative to medium alone. Significant inhibition of growth ($P < 0.05$) is indicated by asterisks. A representative experiment of 5 performed is shown

(Nagashima et al, 1997*a*, 1997*b*). Moreover, co-incubation of carcinoma monolayers with CTLL cells always induced a low level of CTLL growth, as evidenced by increased [$^3$H]-thymidine incorporation (Nagashima et al, 1997*a*,*b*). Also, IL-2 expression on the surface of tumour cells was detectable by flow cytometry (Lin et al, 1995). In addition to IL-2, immunoperoxidase staining has shown the presence of abundant IL-2Rβ and γ in the cytoplasm of carcinoma cells (Figure 2 B, C). Taken together, these data indicate that although the components of the IL-2 pathway are present in carcinoma cells, endogenous IL-2 is not actively secreted but rather utilized by these cells.

### Effects of Abs to IL-2 or IL-2R on growth of tumour cells

To begin to study the role of the IL-2 pathway in tumour cells, we examined effects of various Abs to IL-2 or the IL-2R chain on growth of tumour cell lines. As shown in Figure 3, Abs to IL-2 did not inhibit growth of parental HR, PCI-1 or PCI-13 cells, which do not secrete IL-2. In these experiments, tumour cell monolayers were incubated in the presence of different concentrations of the anti-IL-2 Abs for 3 days, and their growth was assessed in MTT or [$^3$H]-thymidine incorporation assays. These experiments, repeated at least three times with all three tumour cell lines were consistently negative and indicated that growth of tumour cells, which did not secrete measurable levels of IL-2, could not be inhibited by Abs to IL-2.

In a separate series of experiments, we showed that Abs to the binding site for IL-2 on the IL-2Rβ chain, such as TU27 or Mik-β2, used at the concentration > 0.05 μg ml$^{-1}$ significantly inhibited ($P < 0.01$) tumour cell growth (Figure 4). Isotype control Abs used in all blocking experiments did not inhibit tumour cell growth. Abs to epitopes on IL-2Rβ chain not involved in ligand binding (e.g. Mik β3 or Ab 341) were not growth inhibitory (data not shown).

Not only Abs to the IL-2 binding site, but also exogenous rIL-2 used at concentrations > 0.005 nM was found to inhibit growth of tumour cell lines (Figure 5). These results taken together with our previously reported data of in vivo growth inhibition by rIL-2 (Rabinowich et al, 1992; Weidmann et al, 1992; Yasamura et al, 1994), indicate that both the Abs to the IL-2 binding site on the

 © 1999 Cancer Research Campaign

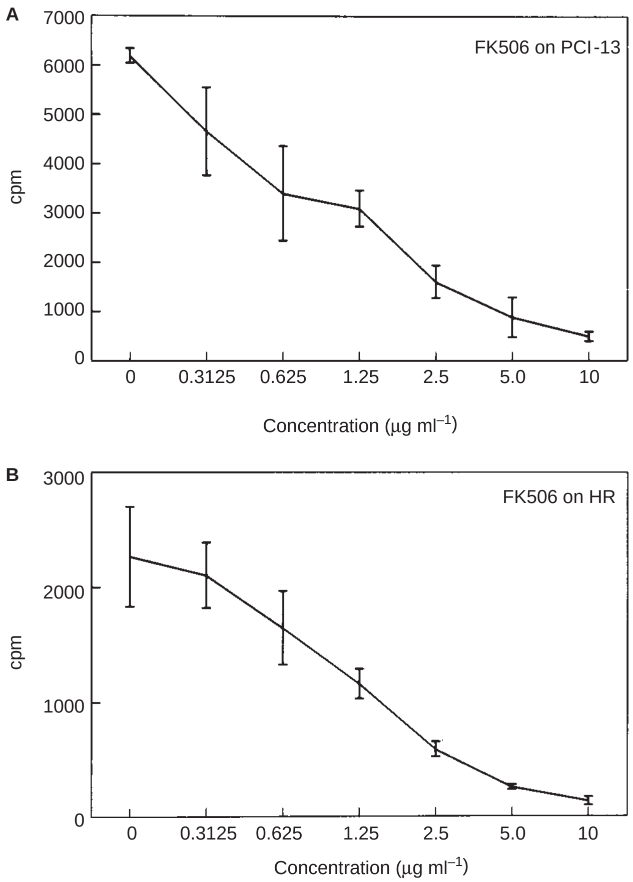

**Figure 6** Inhibition of proliferation of tumour cells (HR in **A** or PCI-13 cell lines in **B**) in the presence of various doses of FK506. Growth was measured in 24 h [$^3$H]-TdR incorporation assays. The data are means ± s.d. of four replicates. One representative experiment of at least 3 performed with each tumour cell line is shown. In all cases, growth inhibition was significantly inhibited ($P < 0.05$)

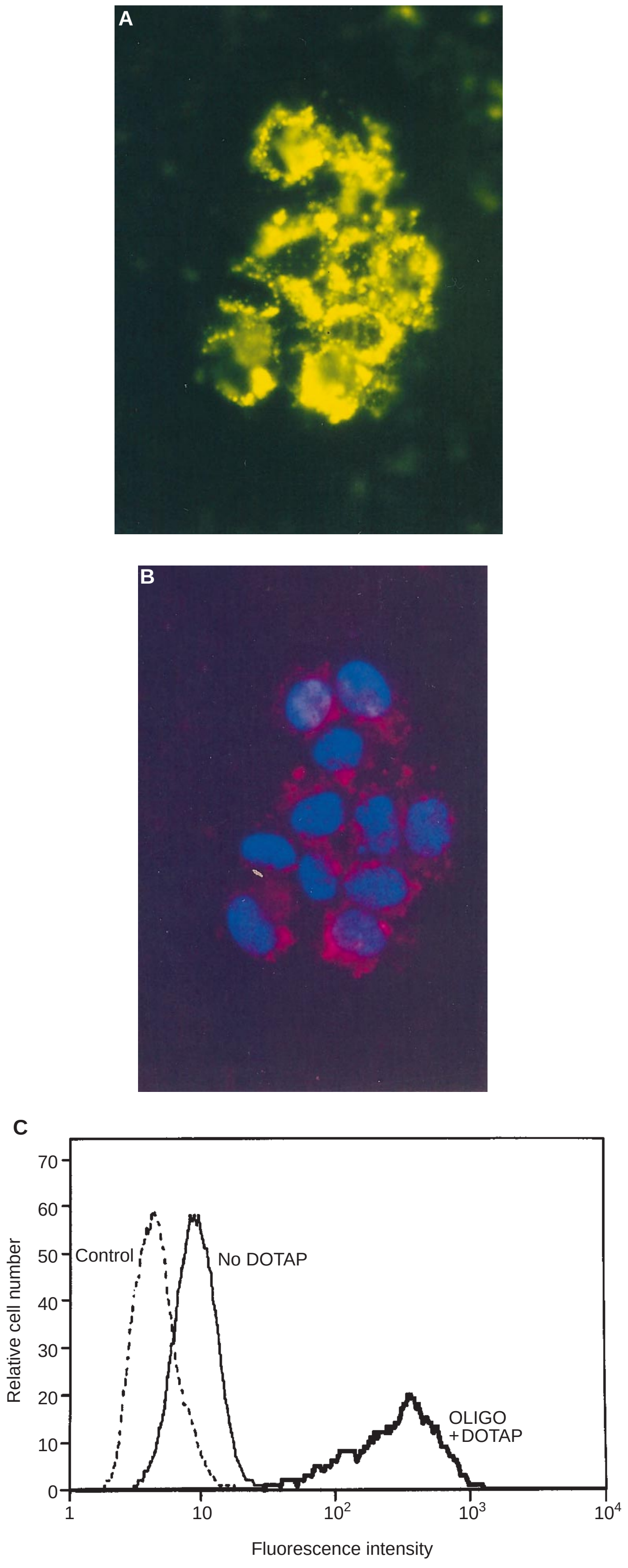

**Figure 7** PCI-1 cells incubated with the IL-2-specific fluorescein-labelled ASO plus DOTAP for 24 h. (**A**) Intracytoplasmic accumulation of AS oligonucleotide plus DOTAP in tumour cells, as determined by flow cytometry. (**B**) Immunofluoressence to show fluorescein-labelled ASO plus DOTAP in the cytoplasm of tumour cells. (**C**) Cy-labelled ASO + DOTAP is shown in the cytoplasm (red). Tumour cells were stained with the Hoechst dye to visualize nuclei. Mag × 400 for **B** and **C**

IL-2Rβ chain and exogenous IL-2 at high molar concentrations can induce a growth-inhibitory signal in tumour cells, possibly through cross-linking of the IL-2R.

In contrast to the effects of exogenous IL-2 on tumour cell growth reported above, IL-2 made by tumour cells appeared to enhance their cell cycle progression (Reichert et al, 1998*a*). The presence in the cytoplasm of these tumour cells of both IL-2 and IL-2R was demonstrated in immunostaining (data for IL-2R are not shown), suggesting that the intracrine IL-2 pathway was operative in tumour cells. Furthermore, this pathway seemed to have opposite biological effects from that driven by exogenous IL-2.

### Effects of cyclosporin A, FK 506 or rapamycin on growth of tumour cell lines

In view of the preliminary evidence indicating that the IL-2/IL-2R complex may play a role in tumour cell growth, we next attempted to interfere with the IL-2 pathway in tumour cells and observe effects on growth. CsA and FK506 are known to inhibit activation of the IL-2 gene transcription in T-cells (Schreiber, 1991). Rapamycin, despite its structural similarity to FK506, has no effect on IL-2 production, but it potently inhibits autocrine/paracrine responses of T-cells to IL-2 and, thus, the progression from G1 to S phase in activated T lymphocytes

*© 1999 Cancer Research Campaign*

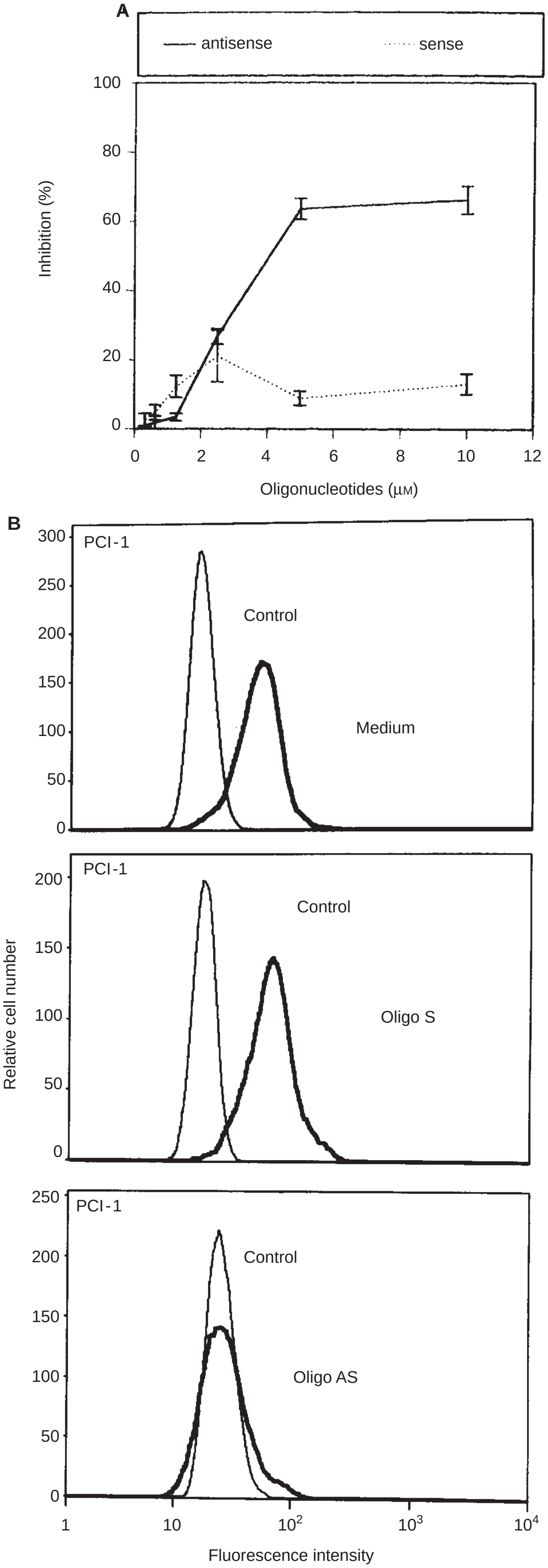

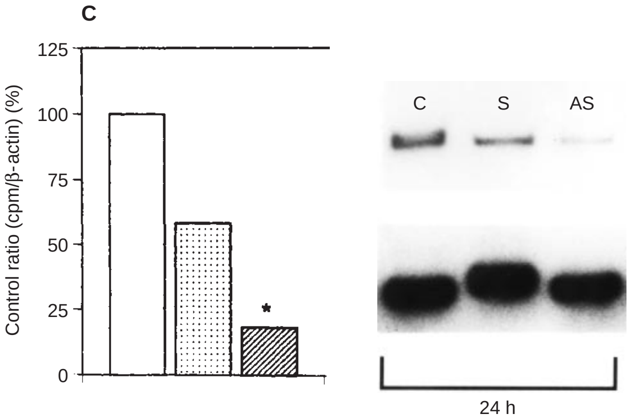

**Figure 8** Growth inhibition of a representative tumour cell line (PCI-1) in the presence of the IL-2-specific ASO. (**A**) Tumour cells cultured in 1% FCS were incubated in the presence of the IL-2 specific ASO or SO for 48 h. Growth was measured in MTT assays and the *P*-value for growth inhibition was $P < 0.0001$. The data are representative of three experiments performed and are mean values ± s.d. (**B**) Flow cytometry with permeabilized cells demonstrates the near absence of IL-2 protein in tumour cells incubated in the presence of the IL-2-specific ASO for 48 h, as compared to SO or isotype controls. (**C**) RT-PCR for IL-2 mRNA was performed in tumour cells incubated in the presence of the ASO or SO (10 µM). Total RNA was obtained from the cells 24 h after the start of the experiment. β-actin was used as a housekeeping gene control (see arrow). The percent inhibition of the ratio of cpm/β-actin determined in Phosphorimager for each lane is shown on the left. The asterisk indicates significant inhibition relative to control at $P < 0.001$

(Schreiber, 1991; Terada et al, 1993). In preliminary studies we observed that tumour cells incubated in the presence of CsA or FK506 down-regulated expression of intracytoplasmic IL-2. We, therefore incubated tumour cells with different concentrations of these drugs in order to examine their effects on tumour cell growth. As shown in Figure 6, FK506 was growth inhibitory to tumour cells in culture. Similar results were obtained with CsA and RPA (data not shown). To minimize possible toxic drug-induced effects on these cells, the dose of each drug inducing close to 50% of growth inhibition was selected for further studies. At these selected drug doses, we examined the short-term effects (24 h) as well as the long-term effects (72 h) of the drugs on growth of tumour cells. As shown in Table 1, CsA at the doses of 1 µg $ml^{-1}$ and FK506 at the dose of 0.5 g $ml^{-1}$ inhibited growth (up to 45%) of the tumour cell lines after 24 h ($P < 0.05$) as well as 72 h ($P < 0.05$) of incubation. The growth-inhibitory effect of these drugs was often greater after 24 h than after 72 h incubation. Rapamycin at the dose of 10 ng $ml^{-1}$ had very little effect on tumour cell growth after 24 h (not significant), but it induced a significant ($P < 0.05$) reduction in growth after 72 h (Table 1). These results were not merely a manifestation of drug toxicity on tumour cells, because in the presence of 200 pM IL-2, drug-induced growth inhibition was significantly reversed (data not shown). Overall, the data suggest that drugs which interfere with the IL-2 pathway in tumour cells also inhibit their growth in culture.

### Effects of IL-2-specific antisense oligonucleotides on IL-2 expression and tumour cell growth

To specifically interfere with the IL-2 pathway in tumour cells, we designed antisense oligonucleotides (as well as sense oligonucleotides to be used as controls), which could bind to IL-2 mRNA and specifically inhibit its translation. A computer program, Oligo

 © 1999 Cancer Research Campaign

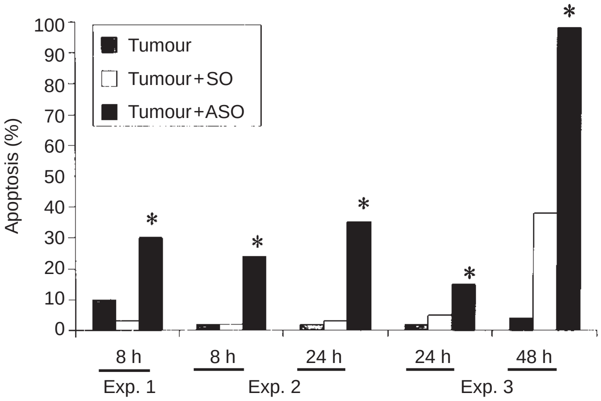

**Figure 9** Increased apoptosis of PCI-13 (experiment 1) or PCI-1 cells (experiments 2 and 3) incubated in the presence of IL-2-specific ASO for 8–48 h. As controls, the same cells were incubated in medium alone or in the presence of IL-2 SO. TUNEL assays were performed to measure DNA fragmentation by flow cytometry. The asterisks indicate a significant difference ($P < 0.001$) from controls, including the SO control. The experiments were performed under conditions described in Materials and Methods

Primer Analysis Software (National Biosciences, Inc., Plymouth, MN, USA), was used to narrow the oligonucleotide selection to those in the 5′ coding region of IL-2. To optimize conditions for treatment of the carcinoma cell lines, the selected oligonucleotides were phosphorothiolated, purified by column chromatography and labelled with fluorescein for preliminary experiments. Next, a DOTAP preparation was selected, which was most likely to facilitate entry of oligonucleotides into tumour cells. Using fluorescein-labelled oligonucleotides, we showed that they accumulated in the cytoplasm of tumour cells after 24 h incubation (Figure 7 A–C). In contrast, only minimal internalization of the oligonucleotides occurred in the absence of DOTAP (Figure 7A). The DOTAP was tested at various concentrations to determine that which was not toxic to tumour cells following cellular uptake (data not shown). Finally, using unlabelled antisense (ASO) and sense (SO) oligonucleotides at the concentration range of 0.3–10 μM, we demonstrated that cultured tumour cells incubated in the presence of the IL-2-specific ASO, but not SO, for 48 h were significantly inhibited in growth ($P < 0.0001$; Figure 8A). Flow cytometry demonstrated that expression of intracytoplasmic IL-2 protein was nearly completely abolished following ASO treatment (Figure 8B). Expression of mRNA for IL-2 was significantly decreased at 24 h after treatment with the IL-2 ASO in PCI-1 cells (Figure 8C). These experiments demonstrated that endogenous IL-2 in tumour cells plays a role in tumour cell proliferation.

### Effects of IL-2 specific antisense oligonucleotides on tumour cell apoptosis

The inhibition of tumour cell growth in the absence of endogenous IL-2 expression could be due to an increase in the rate of cell death. We performed a series of experiments to determine the effects of IL-2-specific ASO on tumour cell apoptosis. As compared to untreated controls and to cells treated with the corresponding SO, tumour cells incubated with the IL-2-specific ASO were significantly ($P < 0.001$) more susceptible to DNA fragmentation in the absence of endogenous IL-2 (Figure 9). The effects of ASO on tumour cell apoptosis were optimal in 8-h cultures with PCI-13 and in 24- to 48-h cultures with PCI-1 (Figure 9). These results suggest that endogenous IL-2 modulates tumour cell growth by protecting them from apoptosis.

### DISCUSSION

The presence of IL-2R on the surface of carcinomas and their ability to synthesize IL-2 suggest that the IL-2/IL-2R pathway is active in human tumour cells and is involved in tumour cell growth. Another indication for the existence of an active IL-2 pathway in human carcinomas is the observed inhibition of tumour cell growth by Abs to the IL-2 binding site on the IL-2Rβ chain expressed on these cells. The two such Abs we used, TU27 and Mik-β2, significantly decreased tumour cell proliferation, when used at sufficiently high concentrations. In previously reported experiments, these Abs were shown to compete with radioactively labelled exogenous IL-2 for binding to the IL-2R (Weidmann et al, 1992). Both exogenous IL-2 at high (nM or μM) concentrations and TU27 or Mikβ2 Abs induced an inhibitory growth signal upon binding to IL-2R on carcinoma cells. In contrast, anti-IL-2R Abs, which recognize the β chain but do not interfere with IL-2 binding, had no effect on tumour cell proliferation. Thus, cross-linking of the IL-2Rβ binding sites for IL-2 on tumour cells by the exogenous ligand available at high concentrations or by Abs to its binding site leads to negative signalling for growth. The mechanism utilized by this growth inhibitory signal is not clear, but we have reported earlier that the absence or low expression of the common JAK3 in HR as well as JAK3 B or M variants associated with the γ chain in SCCHN cells could contribute to altered downstream signalling mediated via the IL-2Rβγ in tumour cells (Suminami et al, 1998). While cross-linking of IL-2R induces a negative growth signal, endogenous IL-2 utilized by the tumour cell in picomolar concentrations via the intracrine or juxtacrine pathway seems to be necessary for proliferation of these cells.

Several different neutralizing monoclonal Abs or a polyclonal Ab against IL-2 that we evaluated had no effect on tumour cell growth. These observations can be explained by the inability of anti-IL-2 Abs to neutralize endogenous IL-2, which may only be released by tumour cells in minute quantities and may be largely bound to IL-2Rβ on their surface, as indicated by the flow cytometry data (Lin et al, 1995). It is also possible that endogenous IL-2 combines with IL-2R in the cytoplasm, as both the ligand and receptor are detectable in tumour cells by immunostaining. Antibody blocking studies, in which the same carcinoma cells transduced with the IL-2 gene and secreting IL-2 were used, confirmed that the anti-IL-2 Abs were able to neutralize secreted IL-2 and inhibit, in part, growth of the transfectants (data not shown).

The observed lack of growth inhibition by Abs to IL-2 in tumour cells could be also explained by the ability of carcinomas to produce other growth factors which could occupy the intermediate affinity IL-2R on tumour cells, preventing binding of anti-IL-2 Abs. For example, IL-15 has a tertiary structure similar to that of IL-2 and is known to be able to bind to the β and γ chains of IL-2R and mediate growth-factor activities similar to those of IL-2 (Grabstein et al, 1994; Giri et al, 1995). IL-15 might block access of IL-2 to IL-2R thus preventing receptor triggering. Surprisingly, exogenous IL-15 does not induce a growth signal in these carcinoma cells, possibly because downstream components of the IL-2Rβγ pathway are altered relative to those in T-cells (Suminami et al, 1998). By immunostaining, we have confirmed expression of IL-15 protein in our tumour cell lines (data not shown). It is,

*© 1999 Cancer Research Campaign* 

therefore, possible to speculate that this cytokine displaces IL-2 from its receptor on the tumour cell surface and thus effectively blocks binding of anti-IL-2 Ab to the IL-2/IL-2Rβ complex responsible for signalling. The fluorescence resonance energy transfer (FRET) data reported by Damjanovich and colleagues (1997) strengthen this hypothesis by demonstrating that an altered conformation of the high-affinity IL-2R complex on the cell surface occurs in the presence of IL-15, allowing the IL-15R α chain to complex with IL2R β and γ subunits (Damjanovich et al, 1997).

To further elucidate the biologic significance of the IL-2/IL-2R pathway in carcinomas, we incubated these cells with the well known immunosuppressive agents: cyclosporin A (CsA), FK506 and rapamycin (RPA). CsA and FK506, binding to immunophilins, inhibit transcription of the IL-2 gene and, therefore, production of IL-2 in activated T-cells (Schreiber, 1991). The drugs do not inhibit expression of IL-2R (Kalman and Klimpel, 1983). Incubation of human carcinomas with CsA and FK506 significantly inhibited tumour cell growth in a dose-dependent manner after 24 h, confirming that the immunophilin-calcineurin-dependent IL-2 gene activation pathway, known to be active in T lymphocytes, also operates in tumour cells. The addition of IL-2 to cultures containing these drugs, significantly diminished drug-induced growth inhibition (data not shown). Rapamycin prevents the IL-2-mediated elimination of the $p27^{Kipl}$ cyclin-dependent kinase inhibitor protein and, consequently, the progression from G1 to S phase in activated T lymphocytes (Nourse et al, 1994). In our study, RPA had very little effect on tumour cell growth after 24 h but showed a pronounced growth inhibitory effect after 72 h of incubation. These results indicate that, similar to its effect on PHA-stimulated T-cells (Terada et al, 1993), RPA interferes with the IL-2 pathway in cycling, but not resting tumour cells. Furthermore, inhibition of cell proliferation by RPA could not be reversed by the addition of exogenous IL-2 at various concentrations (data not shown), again in parallel to results obtained with PHA-stimulated T lymphocytes (Terada et al, 1993).

Taken together, our initial results suggested that the endogenous IL-2 pathway in human carcinomas might play an important role in tumour cell proliferation and survival. Furthermore, immunoprecipitation/Western blot experiments showed that IL-2 produced by tumour cells had the same characteristics as IL-2 produced by lymphoid cells. With the identity of tumour-derived and lymphocyte-derived IL-2 confirmed, it became possible to perform a series of in vitro studies with IL-2-specific ASO, attempting to disrupt this endogenous pathway. Specifically, ASO and SO designed to bind to mRNA for IL-2 were generated and delivered to the cytoplasm of tumour cells by lipofectin, using a cationic lipid transfection reagent, DOTAP. It was possible to demonstrate that IL-2-specific ASO treatment temporarily prevented expression of IL-2 protein and IL-2 message in tumour cells, and that it also significantly inhibited growth of these cells. The sense construct, used as control, had no effect on tumour cell growth. In addition, treatment of tumour cells with ASO, but not SO, significantly increased susceptibility of tumour cells to apoptosis. The antisense strategy allowed us to conclude that in human carcinomas, endogenous IL-2 is involved in tumour cell proliferation and also in protection of tumour cells from apoptosis. Together with our other data, demonstrating an association between overexpression of IL-2 protein and decreased expression of $p27^{Kipl}$, a cyclin D inhibitor, during the S phase of the tumour cell cycle (Reichert et al, 1998*a*), the present results provide compelling evidence that in tumour cells, similar to lymphoid cells, endogenous IL-2 is involved in the regulation of cellular division, proliferation and survival.

The evidence for the presence of the endogenous IL-2 pathway in human carcinomas and its involvement in tumour cell survival has important biological implications. Since tumours appear to express endogenous IL-2 in vivo, as our in situ data indicate (Reichert et al, 1998*b*), they are likely to have an active IL-2 pathway. The participation of this pathway in protection of tumour cells from apoptosis may be important in their survival. A possibility thus presents itself for modulating this pathway by various strategies, e.g. by exogenous IL-2, capable of delivering a negative growth signal at high ligand/receptor ratios; immunosuppressive agents such as CsA, FK506 or RPA, or even IL-2-specific ASO (Stein and Cheng, 1993), in an attempt to control tumour cell growth.

### ACKNOWLEDGEMENTS

The authors are grateful to Dr Lina Matera, University of Torino, Torino, Italy, for her help in designing and performing metabolic labelling studies and to Drs Jennifer Rubin-Grandis and Candace Johnson for reading and critical evaluations of the manuscript. This work was supported in part by the NIH grant CA-63513 (TLW). TER was supported by the Deutsche Forschungsgemeinschaft (DFG), Grant Re1155/1-1.

### REFERENCES

Alileche A, Plaisance S, Han DS, Rubinstein E, Mingari C, Bellomo R, Jasmin C and Azzarone B (1993) Human melanoma cell line M14 secretes a functional interleukin-2. *Oncogene* **8**: 1791–1796

Arzt E, Stelzer G, Renner U, Lange M, Muller OA and Stalla GK (1992) Interleukin-2 and interleukin-2 receptor expression in human corticotrophic adenoma and murine pituitary cell cultures. *J Clin Invest* **90**: 1944–1955

Capcacioli S, diPasquale G, Mini E, Mazzi T and Quatrone A (1993) Cationic lipids improve antisense oligonucleotide uptake and prevent degradation in cultured cells and in human serum. biochem. *Biochem Bioplys Res Commun* **197**: 818–825

Chen K, Demetris AJ, VanThiel DH and Whiteside TL (1987) Double immunoenzyme staining method for analysis of tissue and blood lymphocyte subsets with monoclonal antibodies. *Lab Invest* **56**: 114–119

Chomczynski P and Sacci N (1987) Single-step method of RNA isolation by acid guanidinium thiocyanate-phenol-chloroform extraction. *Anal Biochem* **162**: 156–159

Ciacci C, Mahida YR, Dignass A, Koizumi M and Podolsky DK (1993) Functional interleukin-2 receptors on intestinal epithelial cells. *J Clin Invest* **92**: 527–532

Damjanovich S, Bene L, Matko J, Alileche A, Goldman CK, Sharrow S, Waldmann TA (1997) Preassembly of interleukin-2 receptor subunits on resting Kit 225 K6 T cells and their modulating by IL-2, IL-7, and IL-12. A fluorescence resonance energy transfer study. *Proc Natl Acad Sci* **94**: 13134–13139

Degols G, Leonetti JP, Mechti N and Lebleu B (1991) Antiproliferative effects of antisense oligonucleotides directed to the RNA of c-myc oncogene. *Nucleic Acid Res* **19**: 945–948

Giri JG, Anderson DM, Kumaki S, Park LS, Grabstein KH and Cosman D (1995) IL-15, a novel T cell growth factor that shares activities and receptor components with IL-2. *J Leuk Biol* **57**: 763–766

Grabstein KH, Eisenman J, Shanebeck K, Rauch C, Srinivasan S, Fung V, Beers C, Richardson J, Schoenborn MA, Ahdieh M and et al (1994) Cloning of a T cell growth factor that interacts with the beta chain of the interleukin-2 receptor. *Science* **264**: 965–968

Heo DS, Snyderman C, Gollin SM, Pan S, Walker E, Deka RB, Barnes EL, Johnson JT, Herberman RB and Whiteside TL (1989) Biology, cytogenetics, and sensitivity to immunological effector cells of new head and neck squamous cell carcinoma lines. *Cancer Res* **49**: 5167–5175

Hesslan J-MJ, Branellec AI, Pilatte Y, Lang P and Lagrue G (1991) Differentiation between vascular permeability factor and IL-2 in lymphocyte supernatants from

 © 1999 Cancer Research Campaign patients with minimal-change nephrotic syndrome. *Clin Exp Immunol* **86**: 157–162

Kalman VK and Klimpel GR (1983) Cyclosporin A inhibits the production of gamma interferon (IFN-γ) but does not inhibit production of virus-induced interferon α/β. *Cell Immunol* **78**: 122–129

Lin WC, Yasamura S, Suminami Y, Sung MW, Nagashima S, Stanson J and Whiteside TL (1995) Constitutive production of IL-2 by human carcinoma cells, expression of IL-2 receptor, and tumor cell growth. *J Immunol* **155**: 4805–4816

McMillan DN, Kernohan NM, Flett ME, Heys SD, Deehan DJ, Sewell HF, Walker F and Eremin O (1995) Interleukin-2 receptor expression and interleukin-2 localization in human solid tumor cells in situ and in vitro: evidence for a direct role in the regulation of tumor cell proliferation. *Int J Cancer* **60**: 766–772

Nagashima S, Kashii Y, Reichert TE, Suminami Y, Suzuki T and Whiteside TL (1997*a*) Human gastric carcinoma transduced with the IL-2 gene: Increased sensitivity to immune effector cells in vitro and in vivo. *Int J Cancer* **72**: 174–183

Nagashima S, Reichert TE, Kashii Y, Suminami Y, Chikamatsu K and Whiteside TL (1997*b*) *In vitro* characteristics of human squamous cell carcinoma of the head and neck cells engineered to secrete interleukin-2. *Cancer Gene Therapy* **4**: 366–376

Nakanishi K, Hirose S, Yoshimoto T, Ishizashi H, Hiroishi K, Tanaka T, Kono T, Miyasaka M, Taniguchi T and Higashino K (1992) Role and regulation of interleukin (IL)-2 receptor alpha and beta chains in IL-2-driven B-cell growth. *Proc Natl Acad Sci* USA **89**: 3351–3555

Nourse J, Firpo E, Flanagan WM, Coats S, Polyak K, Lee MH, Massaque J, Crabtree GR and Roberts JM (1994) Interleukin-2-mediated elimination of the p27Kipl cyclin-dependent kinase inhibitor prevented by rapamycin. *Nature* **372**: 570–573

Plaisance S, Rubinstein E, Alileche A, Sahraoui Y, Krief P, Augery-Bourget Y, Jasmin C, Suarez H and Azzarone B (1992) Expression of the interleukin-2 receptor on human fibroblasts and its biological significance. *Int Immunol* **4**: 739–746

Plaisance S, Rubinstein E, Alileche A, Benoit P, Jasmin C and Azzarone B (1993) The IL-2 receptor present on human embryonic fibroblasts is functional in the absence of P64/IL-2R gamma chain. *Int Immunol* **5**: 843–848

Rabinowich H, Vitolo D, Altarac S, Herberman RB and Whiteside TL (1992) Role of cytokines in the adoptive immunotherapy of an experimental model of human head and neck cancer by human IL-2-activated natural killer cells. *J Immunol* **149**: 340–349

Reichert TE, Nagashima S, Kashii Y, Stanson J, Dou QP and Whiteside TL (1998*a*) Interleukin-2 expression in human carcinoma cell lines and its role in cell cycle progression. *Proc AACR* **39**: 152

Reichert TE, Watkins S, Stanson J, Johnson TJ and Whiteside TL (1998*b*) Endogenous IL-2 in cancer cells: a marker of cellular proliferation. *J Histochem Cytochem* **46**: 603–611

Rimoldi D, Salvi S, Hartmann F, Schreyer M, Blum S, Zografos L, Plaisance S, Azzarone B and Carrel S (1993) Expression of IL-2 receptors in human melanoma cells. *Anticancer Res* **13**: 555–564

Sander B, Andersson J and Andersson U (1991) Assessment of cytokines by immunofluorescence and the paraformaldehyde-saponin procedure. *Immunol Rev* **119**: 65–93

Saneto RP, Altman A, Knobler RL, Johnson HM and De Vellis Y (1986) Interleukin-2 mediates the inhibition of oligodendrocyte progenitor cell proliferation in vitro. *Proc Natl Acad Sci* USA **83**: 9221–9225

Schreiber SL (1991) Chemistry and biology of the immunophilins and their immunosuppressive ligands. *Science* **251**: 283–287

Shimizu Y, Weidmann E, Iwatsuki S, Herberman RB and Whiteside TL (1991) Characterization of human autotumor-active T cell clones obtained from tumor-infiltrating lymphocytes in liver metastasis of gastric carcinoma. *Cancer Res* **51**: 6153–6162

Stein CA and Cheng YC (1993) Antisense oligonucleotides as therapeutic agents – is the bullet really magical? *Science* **261**: 1004–1–12

Suminami Y, Kashii Y, Law JC, Lin W-C, Stanson J, Reichert TE, Rabinowich H and Whiteside TL (1998) Molecular analysis of the IL-2 receptor β chain gene expressed in human tumor cells. *Oncogene* **16**: 1309–1317

Taniguchi T and Minami Y (1993) The IL-2/IL-2 receptor system: a current overview. *Cell* **73**: 5–8

Terada N, Lucas JJ, Szepesi A, Franklin RA, Domenico J and Gelfand EW (1993) Rapamycin blocks cell cycle progression of activated T cells prior to events characteristic of the middle to late G1 phase of the cycle. *J Cell Physiol* **154**: 7–15

Waldmann TA (1991) The interleukin-2 receptor. *J Biol Chem* **266**: 2681–2684

Weidmann E, Sacchi M, Plaisance S, Heo DS, Yasamura S, Lin WC, Johnson JT, Herberman RB, Azzarone B and Whiteside TL (1992) Receptors for interleukin-2 on human squamous cell carcinoma cell lines and tumor in situ. *Cancer Res* **52**: 5963–5970

Whiteside TL, Sung M-W, Nagashima S, Chikamatsu K, Okada K and Vujanovic NL (1998) Human tumor antigen-specific T lymphocytes and IL-2 activated natural killer (A-NK) cells: Comparisons of antitumor effects in vitro and in vivo. *Clin Cancer Res* **4**: 1135–1145

Yasumura S, Lin WC, Weidmann E, Hebda P and Whiteside TL (1994) Expression of interleukin-2 receptors on human carcinoma cell lines and tumor growth inhibition by interleukin-2. *Int J Cancer* **59**: 225–234

© 1999 Cancer Research Campaign
